# BUILDING STUDENTS’ CAREER SKILLS THROUGH AGE-FRIENDLY INTERGENERATIONAL CLASSROOM ACTIVITIES

**DOI:** 10.1093/geroni/igad104.0470

**Published:** 2023-12-21

**Authors:** Kimberly Farah, Joann Montepare, Charlotte Frazier

**Affiliations:** Lasell University, Newton, Massachusetts, United States; Lasell University, Newton, Massachusetts, United States; Lasell University, Auburndale, Massachusetts, United States

## Abstract

Changing age demographics are reshaping societies and challenging institutions of higher education to consider how they can respond to age-diverse populations through new approaches to teaching, research, and community engagement. As well, institutions are facing a range of challenges as they look to respond to the contemporary needs of students who will be entering an age-diverse workforce. The pioneering Age-Friendly University (AFU) initiative, endorsed by GSA’s Academy for Gerontology in Higher Education (AGHE), offers a framework within which institutions can begin to address these issues through more age-friendly programs, practices, and partnerships. Drawing on the AFU principle that advocates for promoting intergenerational learning, this presentation will describe several intergenerational classroom activities that have been developed to support students’ success in the workplace – including mock interview sessions, career roundtable discussions, research mentoring efforts, and Careers in Age Week activities. In addition to showing the value of intergenerational exchange to enhance students’ career readiness, these efforts demonstrate how older adults can serve as teaching allies and support the educational mission of higher education.

